# Experiences of violence while in insecure migration status: a qualitative evidence synthesis

**DOI:** 10.1186/s12992-024-01085-1

**Published:** 2024-11-23

**Authors:** Alexandria Innes, Annie Bunce, Hannah Manzur, Natalia V. Lewis

**Affiliations:** 1 City St George’s, University of London, Northampton Square, London, EC1V 0HB UK; 2 University of Bristol, Canynge Hall, 39 Whatley Road, Bristol, BS8 2PS UK

## Abstract

**Background:**

The global movement of people in the context of strict immigration laws and policies places significant numbers of people in insecure migration status worldwide. Insecure status leaves people without recourse to legal, governmental or social protection from violence and abuse. This review synthesized qualitative studies that reported how migrants associated physical and physically enforced sexual violence they experienced with their insecure migration status.

**Methods:**

We conducted a qualitative evidence synthesis of 31 studies published between 1 January 2000 and 31 May 2023, with data from Europe, North America, East Asia, South Asia, the Middle East and Africa. Our thematic synthesis produced 14 inductive descriptive codes, four descriptive themes and three analytical themes.

**Results:**

We generated robust qualitative evidence showing that women experienced sexual violence while in transit or without status in a host state, and that they associated that violence with their insecure migration status. This was the case across the various geographic routes and destination countries. We found evidence that women associated intimate partner violence with lacking (legal) access to support because of their insecure migration status. We found evidence that women connected their unwillingness to leave violent circumstances, and therefore their prolonged or repeated exposure to violence, with a fear of immigration removal produced by their insecure migration status.

**Conclusion:**

To protect people in insecure migration status from experiencing violence that they associated with their migration status, it’s necessary to ensure that the reporting of violence does not lead to immigration enforcement consequences for the victim.

**Supplementary Information:**

The online version contains supplementary material available at 10.1186/s12992-024-01085-1.

## Introduction

Violence is a major public health issue [1]. There is evidence that it has significant long-lasting physical and psychological effects [2]. Moreover, there is evidence that violence is significantly related to social inequality. Existing studies have found links between violence and gender [2–4], ethnicity, place of residence and socioeconomic status [4, 5].

Although economic globalization impacts trade, goods, and services, the movement of people has been increasingly restricted since the 1990s [6, 7]. The number of people globally who live with insecure migration status is difficult to estimate, but includes people worldwide undertaking irregular journeys and crossing international borders without authorization, people living without the correct immigration documentation, and people in temporary or dependent statuses in destination countries [8]. While migration is relatively stable as a proportion of the global population, migrant numbers are increasing as the global population increases, and data on migrant deaths in transit shows that irregular journeys are dangerous and often violent. Insecure migration status leaves people without recourse to legal, governmental, and social protection [9, 10]. Existing research suggests that people in insecure migration status are particularly vulnerable to violence, for several reasons. These include lacking recourse to report violence [11], unregulated transit [12], lack of accountability for violence against people who are outside of their state jurisdiction [13, 14], hostile immigration policies [15, 16], complex immigration policies that make status and associated rights unclear [17], and a lack of knowledge about administrative structures in host countries [18, 19].

To date there has been no study that evidences the phenomenon of violence against people in insecure migration status as a single group, or that synthesizes common experiences of violence amongst that group. Our prior research has offered a conceptualization of insecure status [8] and a systematic review and meta-analysis of the prevalence of violence, operationalizing this conceptualization of insecure status [20]. This systematic review found that the prevalence estimate of physical violence against people in insecure status was 31.16% (95% confidence interval (CI) 25.62–36.70). There was no significant difference in the prevalence estimates among subgroups (gender, status type, timing of violence, geographic region, and perpetrator). Violence was associated with various types of immigration: (1) a lack of status such as people who are residing or travelling without documents; (2) asylum seekers and refugees who self-defined as such; (3) spousal visas; (4) employment-based visas where the status was tied to the employer.

The cycle of violence for people in insecure migration status is built around a threat of state violence in the form of the acts of immigration enforcement such as pushbacks and arrests, detention, violence that might occur in detention settings, the use of restraint, forced removals, and removals to unsafe locations. The threat of this violence drives people in insecure statuses to mistrust state authorities and avoid contact with them [6, 15]. The threat of violence from the state also creates mistrust towards any organization such as health services or specialist services that might be compelled to report client data to immigration enforcement authorities [11, 21, 22]. This means that most of the points of intervention that are available for citizens experiencing violence in the community or in a domestic setting are not available to migrants in insecure status, who are unlikely to avail themselves of these services even when experiencing violence. There is evidence to suggest that this does not just pertain to migrants without status but also to people in any type of insecure status, including asylum seekers [23–25], people on spousal visas, or with ‘no recourse to public funds’ or equivalent [26–29], and people in statuses tied to their employer [25, 30].

While research investigates the relationship between violence and certain categories of immigration status, the particular patterns of violence linked to specified immigration statuses, or occurring generally across types of insecure status have not been substantially explored. We aimed to systematically review studies that reported a perceived association between the experience of being in an insecure status, and the type of violence experienced. The focus of the analysis was on how individuals characterized the link between experiences of violence and experiences of migration-related insecurity.

## Methods

We conducted a thematic synthesis [31] of qualitative studies that reported experiences of violence by people in insecure migration status. This report follows the Cochrane guidance for undertaking a systematic review [32] and the Preferred Reporting Items for Systematic Reviews and Meta-Analyses (PRISMA) reporting checklist [33]. The protocol was prospectively registered on PROSPERO [CRD42021268772] [34]See Appendix 1.

### Eligibility criteria

We included primary studies of any design that used qualitative methods for data collection (e.g., interview, focus group, observation, document review) and analysis (e.g., content, narrative, discourse, thematic, grounded theory), if they documented first person excerpts that described an association between insecure migration status and the experience of violence. Only peer reviewed reports in English published since 1 January 2000 were included.

This review followed a Population-Exposure-Outcome (PEO) design, in which the exposure was insecure status, and the outcome was violence. All participants were migrants who were in a status that embedded a form of insecurity. All participants experienced violence in the context of insecure migration status. To meet the inclusion criteria, the violence had to be linked to the insecure migration status. The analysis traced the link that participants made between the violence they experienced and their insecure migration status. Insecure status was defined according to Innes 2023 [8] and was formed of a spectrum of different statuses (see Appendix 2). These included undocumented, asylum seeking, family-based and employment-based statuses.

The definition of physical violence that was adopted in this study included interpersonal and state physical violence and physically forced sexual violence (rape, sexual assault) as specified in the World Health Organization definition and typology of violence [35]. We included all forms of physical and physically enforced sexual interpersonal violence. We also included state violence, where physical violence and/or physically forced sexual violence was perpetrated by an agent of the state acting in their professional capacity (including border enforcement, police, and immigration officers). The focus on physical and physically enforced sexual violence was not intended to undermine the relevancy of other forms of violence such as structural, systemic, legal, biological, psychological, and emotional. Rather, it was to limit an unwieldy study to the most explicitly violent contexts to offer insight into where physical violence is experienced as linked specifically to insecure migration status.

### Search strategy

We combined three concept clusters that were reviewed by the team of researchers, which included expertise in migration studies, violence, and research methods. The concepts clustered terms relating to ‘immigration’, ‘violence’ and ‘methods’. A Boolean search was carried out to link each of the concept clusters with each other (AND search) while using multiple descriptive terms in each of the three clusters (OR search). See Appendix 3 for more details.

Database selection was based on initial scoping, combined with areas of expertise across the authorship. Five databases were selected: *Embase*, *Social Policy and Practice*, *Political Science Complete*, *SocINDEX* and *Web of Science Social Sciences Citation Index*. We ran the searches on 22 September 2021 and updated on 31 May 2023, for records from 1 January 2000. The start date was chosen to exclude work that predated immigration reforms in the 1990s. All selected studies were subject to backwards and forwards citation tracking to identify additional studies for inclusion. Forwards citation tracking was carried out using the tool available in *Google Scholar*.

### Study selection and data extraction

Endnote was used to deduplicate the search results and to save PDF files. The first reviewer screened all titles and abstracts against the inclusion and exclusion criteria and studies that satisfied the inclusion criteria at the abstract stage then went forward to full text screening. Full texts were screened against the exclusion matrix (see Appendix 4) and the reason for exclusion was recorded. Both stages of screening were carried out in Rayyan [36]. The second reviewer independently screened 20% at both stages of review and all discrepancies were resolved through discussion and consensus.

### Data collection process

Details of each included text were recorded in a bespoke Excel table documenting seven categories: (a) report ID and year, (b) insecure status type, (c) violence type, (d) dataset details, (e) country or region of violence, (f) participant characteristics, and (g) notes. These details were documented by the first reviewer and then checked for accuracy by the second reviewer.

### Quality assessment

We carried out a detailed risk of bias assessment of each included study using the Critical Appraisal Skills Programme (CASP) Checklist for Qualitative Research (CASP 2018). We assessed quality for ten domains per study: (1) Was there a clear statement of aims? (2) Is qualitative methodology appropriate? (3) Was the research design appropriate for the aims? (4) Was the recruitment strategy appropriate for the aims? (5) Was the data collected in a way that addressed the research issue? (6) Was the relationship between researcher and participants considered? (7) Were ethical issues considered? (8) Was the data analysis sufficiently rigorous? (9) Was there clear evidence of findings and (10) Is the research valuable? We did not give the study an overall score but reported the complete assessment (see Appendix 5). The quality assessment was carried out independently by two reviewers and any disagreements were discussed, resolved, and recorded.

### Synthesis

We adopted a thematic synthesis approach as the most suitable for our research question exploring experiences of violence [31]. All the included reports were imported into NVIVO.

At stage one, first reviewer used a combination of inductive and deductive approaches to code each report line-by-line. The coding strategy was derived through an iterative process after two readings of the included reports. Only first-person descriptions of physical violence that were linked to insecure migration status specifically by the speaker were coded. The link might have been made in contextualized information provided in the article (such as the author stating that they asked the speaker specifically about their insecure migration status). These codes were reviewed by and agreed with the second reviewer who coded 100% of reports. All reports were double coded by the first reviewer to ensure any codes derived through line-by-line coding were assessed for every report, and coded once by the second reviewer. Discrepancies were logged in an Excel table, discussed, and agreed upon.

At stage two, the first reviewer developed analytical themes by reviewing co-occurrence across inductive and deductive codes. The two reviewers discussed the themes before finalizing.

### Findings

#### Characteristics of the included studies

**Fig. 1:**
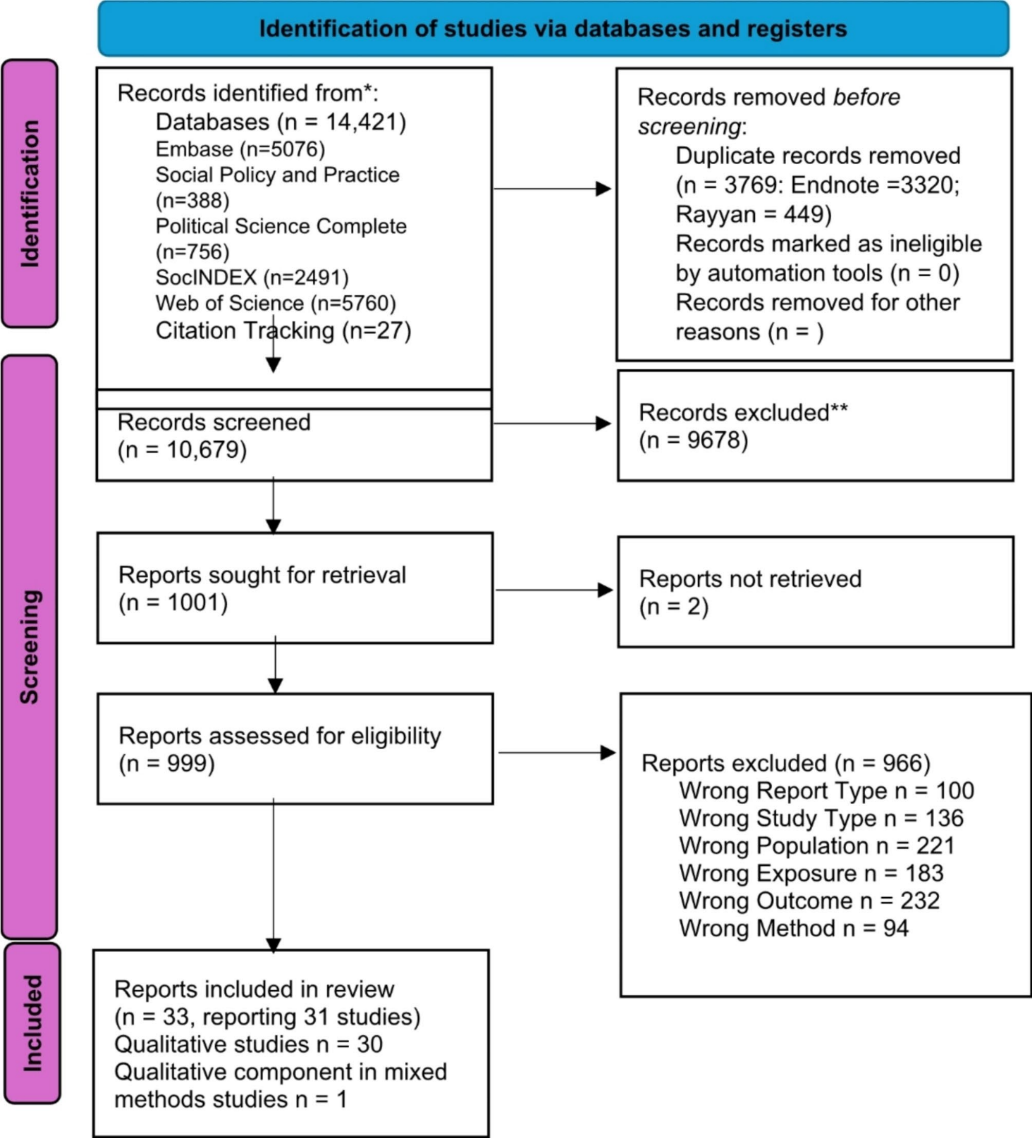
Prisma flow diagram

We included 31 studies, published in 33 reports, reporting qualitative experiential data of a total of 1507 migrant participants (at least 49% female, two studies did not disclose the gender distribution) (Fig. 1 and Table 1). All but two [24, 37] of the studies used a form or a combination of forms of interview methodology. Six studies used ethnographic or participant observation [23, 38–42], three studies used focus groups [18, 40, 43, 44], and three used participatory action research [27, 37, 45].

**Table 1:** Characteristics of included studies

Study ID	Insecure status type	Violence type	Region of violence	Dataset details	Population
Adeyinka 2023	Undocumented	Sexual violence	Niger, Libya, Italy	Semi-structured interviews	31 Female Nigerians
Anitha 2008	Spousal visa, NRPF	Domestic violence, IPV	UK	Semi-structured interviews	30 South Asian women
Anitha 2010	Spousal visa, NRPF	Domestic violence, IPV	UK	Participatory Action research and semi structured interviews	30 Indian, Bangladeshi and Pakistani women with NRPF
Baird 2014	Refugee / undocumented	Physical violence	Europe	Participant observation / semi-structured interview	40 respondents (73% male), of Somali, Sudanese, Eritrean, Senegalese and Nigerian origin who had used a smuggler to cross borders.
Bhatia 2019	Asylum seekers	State violence	UK	Ethnographic observation, in-depth interviews.	22 asylum seekers and undocumented migrants.
Boyd 2019	Undocumented	Sexual violence (rape)	South Africa	Interviews	4 migrant women victims of sexual violence.
Critelli & Yalim 2020	Refugee / undocumented	Domestic violence, IPV	USA	Interviews	25 refugee domestic violence survivors.
Erez et al. 2009	Spousal	Domestic violence, IPV	USA	Interviews	137 immigrant women who had sought advice from domestic violence specialist service providers.
Erez & Bach 2003	Spousal	Domestic violence, IPV	USA	In-depth interviews	10 foreign-born immigrant women married to US military servicemen.
Fennig & Denov 2022	Refugee, undocumented	Physical violence	Israel, in transit (north and northeast Africa, Sinai)	In-depth interviews	34 Eritrean refugees (56% female) seeking asylum in Israel.
Gabreyesus et al. 2018	Asylum seekers	Sexual violence	Israel	In-depth interview and focus group	12 in depth interviews (6 male, 6 female) and 8 gender-segregated focus group discussions with Eritreans of reproductive age in Israel
Gabreyesus et al. 2019	Asylum seekers	Sexual violence	Israel, in transit	In-depth interview and focus group	12 in-depth interviews and 8 focus groups with Eritreans in Israel
Grossman-Thompson 2023	Employment-based	Physical violence, from employer	Various (India, Middle East)	In-depth interviews	30 Nepalese female migrant workers
Infante et al. 2020	Undocumented	Physical and sexual violence	Mexico	In-depth interviews	58 (52% male)central American migrants in transit through Mexico.
Jimenez-Lasserrotte et al. 2020	Undocumented	Community violence, sexual violence	Europe	Interviews	26 female international migrants who had crossed the sea in a small boat in the last 5 years.
Keygnaert et al. 2014	Undocumented	Physical violence	North Africa	In-depth interviews conducted by community researchers; community based participatory action research.	154 sub-Saharan migrants (39% female) living irregularly in Morocco.
Kovner et al. 2021	Asylum seekers	Community violence, state violence	Europe (Greece)	Collection and analysis of testimonies published by local and international NGOs	2 testimonies cited; 4 published reports consulted.
Laughon et al. 2022	Asylum seekers	Physical violence, gender-based violence, violence against children	Mexico	Semi-structured interviews	43 female asylum seekers living in an informal camp while in transit through Mexico.
Leyva-Flores 2019	Undocumented	Physical violence	Mexico	In-depth interviews	58 migrants (52% male) who had experienced violence while in transit through Mexico.
Liang 2023	Spousal, trafficking	Domestic violence, IPV	China-Vietnam border	In-depth interviews	10 trafficked women and 17 local residents.
Liversage 2021	Spousal	Domestic violence, IPV	Europe (Denmark)	Dramatized narratives, life narrative interviews.	35 migrant women who had experienced partner abuse.
McMahon & Sigona 2021	Refugee, asylum	Physical violence	Europe	Interviews	500 asylum seekers: 205 in Italy, 215 in Greece, 20 in Malta and 60 in Turkey.
Minaye 2012	Employment-based	Physical violence	Gulf states	Interviews	8 Ethiopian trafficking returnees who had been recruited by force, deception, or in case of extreme vulnerability and experienced slavery-like conditions.
Omar 2022	Undocumented, refugees	Physical violence	East Africa, Middle East	Interviews	8 female migrants who crossed Gulf of Aden from East Africa to Yemen
Pan & Yang 2012	Employment-based	Physical violence	East Asia (Taiwan)	Participant observation and interviews	16 migrant women workers.
Parson 2016	Undocumented	Domestic violence, IPV, gender-based violence	USA	Participant observation, focus groups, life-history interviews	6 focus groups with total of 45 female participants, 10 life history interviews with female victim-survivors.
Radziwinowiczowna 2020	Undocumented	State violence	USA	Interviews	25 Mexican returnees (88% male) who had been detected, detained and deported from the USA to Mexico.
Reina & Lohman 2015	Spousal	Domestic violence, IPV	USA	Interviews and focus groups	10 undocumented Latina migrants from Central and South America.
Reina 2014	Spousal	Domestic violence, IPV	USA	Narrative interviews and focus groups	14 female immigrants from Mexico, Central and South America.
Salcido & Adelman 2004	Undocumented	Domestic violence, IPV	USA	Ethnography, in-depth interviews	10 undocumented immigrant women from Mexico
Sharma 2017	‘Insecure status’	Gender-based violence	UK	Participatory action research	61 women in insecure migration status participating in a pilot project with the ‘Safety for Sisters’ organization.
Valdovinos et al. 2021	Undocumented	Domestic violence, IPV	USA	‘Testimonio’ interview	20 undocumented Latina cisgender immigrants who experienced IPV.
Vidales 2010	Undocumented, spousal	Domestic violence, IPV	USA	Interviews and participant observation	31 female victims of domestic violence without legal resident status.

Ten studies were located in the USA [18, 40–42, 46–50], eight in Europe including the UK [23, 24, 26, 27, 37, 38, 51–53], two in East Asia [39, 54], four in Africa [45, 55–57], four in the Middle East and South Asia [43, 44, 58–60], and three in Mexico [61, 62].

Fifteen of the studies linked violence to undocumented status [38, 40–42, 45, 46, 49–51, 55–58, 61, 63], seven to spousal sponsorship [18, 26, 27, 47, 48, 52, 54, 64], five to asylum seeking [23, 24, 43, 44, 53, 62] and three to employment-based statuses [39, 59, 60]. One text defined immigration status just as ‘insecure’ [37].

#### Descriptions and patterns of violence experienced when in insecure migration status

We developed 14 inductive and 4 deductive codes. The inductive codes linked the types of violence that were most commonly experienced in insecure migration status. The deductive codes summarized the perceived link between violence and insecure status: (1) direct insecurity, (2) fear of removal, (3) lack of recourse to state support (e.g. law, refuge, economic), (4) gender (Appendix 6.

We developed four analytical themes summarizing experiences of violence linked to insecure migration status: *Vulnerability to Sexual Violence*, *Lack of Pathway to Support*, *Power Imbalance* and *Gender: violence against women* (Table 2).

**Table 2:** Themes and codes

Themes	Codes		Total number of references
Vulnerability to Sexual Violence	Direct Insecurity	Sexual violence	73
		Sex-gender	32
		Employment	22
		In transit-highly vulnerable	62
		In destination-highly vulnerable	34
		Community Violence	5
		Racist Violence	4
		State violence	17
Lacking Pathway to Support	Lack Recourse Fear Removal	IPV-unable to leave	7
		Social isolation	3
		Family violence	5
		Fear of child protection	3
		Sexual violence	5
		No access to support	13
Power Imbalance	Fear Removal Direct Insecurity	Family violence	2
		Fear of child protection	2
		Sexual violence	2
		No access to support	1
		State violence	18
Gender: violence against women			132

##### Vulnerability to sexual violence

The analytical theme ‘Vulnerability to Sexual Violence’ was drawn from the data linking physically forced sexual violence to direct experiences of insecurity, that is, the association participants in the included studies made between sexual violence they had experienced that they directly related to their lack of immigration status at the time the violence occurred. In this context, ‘Direct Insecurity’ was the most commonly occurring deductive code, and referred to excerpts that described an exposure to violence that was directly linked to experiences of being in insecure migration status. In this context the overarching insecurity gave rise to violence (rather than the violence being repeated or prolonged as a result of insecurity). This applied primarily to people who were without status and most commonly occurred with the descriptive codes ‘sexual-violence’, ‘in-transit-highly-vulnerable’, ‘in-destination-highly-vulnerable’, and ‘employment-based-violence’. Violence that was associated with the direct insecurity of being without status was primarily evident during undocumented migration journeys, particularly when those journeys were facilitated by smugglers or traffickers. It was also evident after arrival in a destination country if a person remained in undocumented status in the receiving country.

##### Sexual violence on migration journeys

There were many descriptions of physically enforced sexual violence occurring during migration journeys, which were primarily documented in the dataset through African states on journeys towards Europe, through the Sinai desert towards Israel, and through central America and Mexico towards the US. These descriptions emphasised the lack of power to resist sexual violence on the part of migrant victims. For example, one participant recounts “I had no choice but to give in” [55], and another specified “I felt so powerless with the guns pointed at us” [44]. While extreme violence is described in detail across many of the studies, at times, sexual violence was described as a necessity or as a known burden attached to the migration journey. A level of acceptance of the lack of power to resist is indicated, for example, “it was not easy, and it’s life” [45]. At times the violence associated with the migrant journey was expected and described as transactional. This might be in an immediate sense, for survival as recounted in Adeyinka [55], or as a form of payment for the intended end result of a successful migration: “Even migrants say that women have the ticket to transit between their legs, and the police tell them they have to have sex with them to be able to move on” [63], and “Just think of it as paying for protection with [your] body” [61]. Nevertheless, it is worth noting that the reference to a transaction is not denoting consent.

##### Sexual violence at destination

Experiences of psychically enforced sexual violence in the destination country were linked to the vulnerability of being without immigration status that saturates all parts of life. In most descriptions of violence in this context, the violence was not expected after arrival at the destination. For example, “I would cry every time they had sex with me, because that was not what they told me that I would come and do here” [55]. Sexual violence in the destination arose from living quarters or from employment arrangements where women were vulnerable because their insecure status meant that they had little power to resist physical violence [43]. They might have been coerced into sex work in order to pay back high fees for transit, with the threat of physical violence or imprisonment if they refuse. For example, one woman recounted being forced to have sex with men who were brought to her room; she was not allowed out in case she ran away [55].

One article detailed ‘marriage trafficking’ cases where women were sold, against their will, into marriage and trapped in the situation by bearing children. These women also described sexual violence, for example:

“That night, I was raped by a cripple. Later, I found out that I was sold to that cripple (Trafficked woman 4). … I was raped by my husband in his house the first night. Then I was locked up by my husband’s family in a room with a big iron door. (Trafficked woman 5)” [54].

These examples are indicative of the body of excerpts that show the types of violence associated with direct insecurity, conceptualised as a lack of immigration status or an explicitly insecure immigration status. Sexual violence occurred most frequently and co-occurred with descriptions of being in transit and with descriptions of insecurity in the destination country. Direct insecurity and sexual violence can also be linked to the cost of transit and the desire for smugglers or traffickers to recoup the costs, such as in the case of marriage trafficking and forced prostitution. This vulnerability to sexual violence in particular was clearly associated by participants across several studies with their explicitly insecure, undocumented status [38, 39, 43–45, 51, 54, 55, 57, 60, 61, 63].

##### Lack of pathway to support

The deductive code ‘Lack recourse’ occurred mainly with two inductive codes: ‘IPV-unable to leave’, and ‘no access to support’. These co-occurrences were grouped into the analytical theme ‘Lack of pathway to support,’ which referred to circumstances in which exposure to physical violence was prolonged because there was either no recourse to formal support, no means of accessing support that should have existed, or a belief on the part of the victim that they did not have access to any form of support. This theme tended to include primarily spousal visa statuses and intimate partner violence. The focus on family life was evident in the inclusion of descriptive codes ‘family violence’ and ‘fear of child protection,’ which also co-occurred in this category.

It is worth noting that, while there is of course a strong relationship between the codes ‘lack recourse’ and ‘no access to support,’ they were defined differently and were not always co-occurring. ‘Lack recourse’ referred to a legal or official lack of recourse to support, such as experienced by people in undocumented statuses and with No Recourse to Public Funds visa stipulations (or similar by country). ‘No access to support’ referred to social, community and family support. Access was defined more broadly than recourse, in that someone who *did* have recourse to support might still face barriers to accessing it as a result of their insecure migration status. For example, one participant who was experiencing IPV shared her lack of both access and recourse:

“My husband beat me up several times, especially when I gave birth to a baby girl. I never discussed my problems with anyone. Not with my own family, because I was not allowed to take phone calls. When my in-laws turned against my baby—they refused to bring milk and nappies for her—then I decided to leave that house. I had no place to go to, no money for food and no friend or relative or any other person who knew me. I tried several refuges, but they would find out that I am on ‘no recourse’ and they refused to take me.” [27].

The majority of the qualitative studies that were coded in this category were retrospective interviews with women who had eventually left abusive situations. Nevertheless, many included descriptions of being unable to leave violent relationships for a prolonged time, because they lacked the recourse for support. For example, the following quote describes an intervention by social workers that came to nothing. ‘it was summer, and I was wearing shorts, and they could see all the bruises I had all over my body. Regardless of this, they didn’t help me.” [52]. The author then paraphrases that the social worker told the participant that she would be forced to leave Denmark, her destination country, if she divorced her husband. This information later contributed to a suicide attempt by the participant. In this case, the social workers themselves believed the participant to lack recourse and so did not provide it. Thus, while recourse to support should have been provided despite insecure status, the insecure status of the individual led to a lack of access.

Women described their inability to leave and their enforced compliance in abusive situations as linked to their immigration status, stating ‘because I am illegal’ or ‘he used my immigration status against me’ [47], or describing their lack of knowledge as delaying their departure from violent relationships [27, 50]. In these contexts, women were trapped into situations where they were repeatedly subject to violence, and they were vulnerable to violence increasing in severity, because they feared for their immigration status [27, 48, 50, 52]. They lacked either the recourse to police, social services, or healthcare, either as a direct consequence of their insecure status or because they lacked the *knowledge* that they had recourse. In some cases, support was denied because the providers believed that the victim did not have recourse to support, and advised women that leaving an abusive relationship would result in the loss of immigration status [52]. This demonstrates the complexity whereby even if legal recourse is provided for, it is still lacking in a practical or administrative capacity.

Another participant did not have sufficient support to give her confidence that she would not be separated from her children, despite suffering physical, psychological and economic abuse. She stated that she feared that if she asked for help, her children would be removed from her care [46]. The need for types of support that would make seeking help possible is raised in the data. The *belief* in a lack of recourse to support was driven by a lack of access, such as one woman who was advised to attend hospital, but was refused any help to make an appointment or go to the hospital, and was unable to do so alone [52]. Another participant referenced being given pamphlets but no further help and no indication than she could seek support despite lacking a social security number in the US [40].

To summarise, insecure immigration status meant that there was often a disconnection in the chain of support that would allow violence to be reported, and there was often a lack of knowledge about support entitlement. The latter meant that women in insecure status experienced an underlying fear that support seeking would result in a loss of status and so support was not sought or accessible.

##### Power imbalance

The power imbalance embedded in spousal and employment-based visa types can be understood as a significant vector of insecurity. In the included studies, women linked their fear of removal from a country to this embedded power disparity whereby a visa relies on a relationship with a spouse or a particular employer. Women who feared they would be removed from the country described how they remained in violent relationships even after experiencing episodes of physical violence at the hands of their partner. The ‘Fear Removal’ deductive code was most meaningfully connected to the inductive descriptive code ‘IPV unable-to-leave’, which highlighted where migrants associated violence with the fear of being removed from a host country. It was also connected to the inductive descriptive code ‘employment related violence’, whereby a visa tied to a particular employer or agency-facilitated labour migration embeds a power inequality that makes it difficult to leave even when facing physical abuse.

Participants in several studies indicated that they remained in spousal relationships where they experienced physical and/or physically enforced sexual violence because they feared removal from the country would be a consequence of leaving. This fear was reproduced and accentuated in the context of threat from the citizen perpetrator [18, 46, 47, 64]. In these cases, insecure immigration status was not necessarily the initial reason for violence, but violence was prolonged as a result of insecure status because women felt unable to leave the relationship on which their status was based. Immigration status was used as a threat. An excerpt from Anitha [26] suggests that the participant believed this abuse of her insecure immigration status was active and intentional: “once here, I soon came to know that they only wanted a servant for their house” … “My visa expired but (they) were not ready to apply for indefinite leave for me. His mother always used to say, ‘Deport her!’” [26]. In the context of a spousal visa, the applicant must file the paperwork to renew immigration status, and they must supply paperwork to evidence the application. This sort of paperwork can be withheld by an abusive spouse or family member. In the example cited above, the woman was left in insecure status with little recourse to refuse the conditions of servitude and the physical violence imposed upon her due to the power disparity she experienced where her immigration status was controlled by her spouse and his family. She was isolated in her domestic setting, and her vulnerability to violence due to the fear of removal was experienced in the context of both intimate partner and family violence. This key example demonstrates that the power disparity in the relationship was enhanced by lack of immigration status, which was used as an additional intersectional vector of abuse.

A similar power disparity was evidenced in the context of visas tied to a particular employer. The ‘employment-based-violence’ descriptive code also co-occurred with ‘2FEAR-REMOVAL’. This applied whereby women described feeling trapped in conditions of violence in their place of employment [60], or being forced to have sex with an employer under threat of removal [39]. The data showed that when people fear losing their immigration status, they are more likely to remain in violent situations to protect their status, which prolongs exposure to violence [40, 46, 47]. In these contexts, the victims of violence associated their experiences of violence with their insecure migration status.

A more explicit power imbalance was identified in the data in the context of the disparity between the state and undocumented migrants. This falls under the theme of power imbalance but is distinct from the power disparity embedded in spousal and employment visa types. Relevant excerpts referred to police violence, such as “the police beat me and broke my teeth” [38], or the above-cited example of police sexual violence [61]. State violence was also recounted during immigration detention, removal, or in the context of border enforcement [44, 49, 62]. The examples in these cases show explicit abuse of power on the part of officers of the state, and a lack of power to resist on the part of migrants without documents.

##### Gender: violence against women

The theme of gender was supported by 26/33 included reports. Excerpts were coded as gender when the violence was explicitly related to gender, particularly violence against women migrants. Gender did not refer to a particular dimension of migration-related insecurity but co-occurred with 56% of the codes identifying direct insecurity, 71% of the codes identifying a fear of removal, and 51% of the codes identifying a lack of recourse.

Most of the excerpts relating to gender either referred to sexual violence, specifically rape during migration journeys [43, 44, 55, 63], or to [50] domestic violence (IPV and/or family violence) where insecure immigration status was linked specifically to threats of removal and a lack of recourse to support, as discussed above [18, 26, 27, 48, 64].

Additional examples in the ‘Gender’ theme were related to pregnancy and women fearing for their children; for example, threats to take away children [50] or threats related to pregnancy [52]. Pregnancy should be understood as an important intersecting vulnerability, whereby it increases the dependence of women in insecure immigration status on their sponsoring spouse or family, and a child can be used as an additional form of threat and coercion linked to insecure immigration status.

## Discussion

This thematic synthesis included 31 studies with 1507 people who described their experiences of physical and/or physically enforced sexual violence while in insecure immigration status in Europe, North America, East Asia, South Asia, the Middle East and Africa. We developed four analytical themes summarising the contexts that gave rise to violence participants directly associated with their insecure migration status. These were ‘Vulnerability to Sexual Violence’, ‘Lack of Pathway for Support’, ‘Power Imbalance’ and ‘Gender: violence against women’. In what follows we draw out some additional insights on the descriptive code sexual violence, and on the prolonged exposure to violence that is associated with insecure migration status.

### Vulnerability to sexual violence

This study did not code for sexual violence unless *physically forced* sexual violence was specified at the screening stage. The intention was to limit a large, unwieldy study to the most severe and explicit types of violence associated with insecure migration status. Nevertheless, sexual violence was the most frequently recorded descriptive code. This was particularly associated with being directly insecure such as in undocumented status, or when undertaking a migration journey. There is reason to believe that the problem of sexual violence experienced by people in insecure migration status is even more widespread than this data suggests. As Catherine Boyd argues, when migrants are undocumented they are extremely unlikely to report rape (48: 16). Barriers to reporting violence are compounded by being in transit. Migrants undertake risky journeys to reach a final destination, and often once embarked, there is little opportunity to report violence or crime. Where violence can be and is reported, responses to reports of violence are unlikely to be effective. Leyva-Flores et al. [61] evidence the supposition that violence is unlikely to be reported in a mixed-method study: of their total sample population (12,023 migrants in transit), only 13.9% of migrant victims of violence reported that violence to the authorities. When the perpetrators of violence are the same people who migrants in transit are relying on to cross dangerous terrain such as desert and sea, or are state or authority figures, it becomes clear how substantial underreporting is likely to be. There is a clear power disparity between the perpetrators of sexual violence and the migrant victims undertaking undocumented journeys.

Additionally, for migrants in transit there is a risk that the known danger of sexual violence can be conflated with welcoming sexual contact during the migration journey. This might be constructed as a transactional element to sexual violence. This links to the cost-benefit approach to understanding migration decision-making, and encompasses the risk of violence during an undocumented migration journey as part of the ‘costs’ of the migration that have been assessed and accepted by the ‘rational’ migrant [65]. In many cases, women might have been aware of the risk of sexual violence, but this knowledge is located in a broader context of the reasons for the migration and any mitigating precautions the women might have taken. These included travelling in groups, and with men, or paying higher prices for safe transit. For example, Adeyinka [55] documents evidence of failed precautions. The view that sexual violence during a migration journey is transactional relies on a logic of blaming the victim, and should not be sustained [66–68]. Sexual violence should not be reconstructed as consenting in this context. It is likely that data limited to physically forced sexual violence is substantially underreported, because of this conflation between transactional sexual violence and consent.

### Lack of pathway to support

This review found that women are likely to remain in violent relationships due to fear of immigration removal. This of course is not a new finding; indeed, in 1991 Kimberle Crenshaw found that black and Latina women perceived the threat of (immigration) removal to be worse than the threat of violent relationships [69]. Stefani Vasil [17] found that women remain in violent relationships due to perceived visa insecurity, even though that insecurity is not always based on a well-founded fear. For example, in some cases women hold lawful permanent resident status but still fear that they may lose their status if their relationship breaks down [17]. Measures to reduce this phenomenon have been introduced, such as the Violence Against Women Act introducing a petition to remove the two-year relationship condition on spousal visas in the US in cases of documented domestic violence, or the Indefinite Leave to Remain route for domestic violence and abuse in the UK, or the family violence provision in Australia. However, these routes can be viewed as riskier than remaining in a violent relationship, because the applicant must declare the relationship has broken down before her immigration status is secured. The burden of proof of violence required often relies on documented occasions such as encounters with police, which we know immigrant women in insecure status are likely to avoid [8, 11]. It is clear in the findings of this study that, despite pathways to status existing, they were often not known to the women [52], or they seemed too risky to pursue in the first instance [50].

### Power imbalance

Literature has recognised the precarity that is linked to employment-based statuses [25, 30]. The vulnerabilities attached to spousal visas status have also been explored [26–28, 70, 71]. However, considering them together under the common characteristic of an embedded inequality suggests that this power imbalance creates a significant vulnerability that prolongs exposure to violence. The data in this review suggested that violence is experienced as a direct result of the power imbalance embedded in visa types that require a continuous relationship to maintain status. Visa requirements enhance the difficulty of leaving an abusive relationship whether that relationship is with a spouse or an employer because they increase the power of the perpetrator by formalising that power into a legal status that is called to question if the relationship breaks down.

In addition, the findings suggest that state violence against migrants in insecure status is frequently experienced by migrants who have no recourse to report or seek protection from this violence. It is often subsumed under law enforcement or border enforcement, particularly where state violence occurs during the process of apprehension and detention. This sits adjacent to police violence and brutality against racial and ethnic minorities [72], adding the intersection of insecure immigration status as another vector for risk of violence, and lack of ability to resist this violence.

### Gender: violence against women

Gender is an important intersecting vulnerability that impacts women in insecure migration status and marks a significant inequality that is exacerbated by insecure status and violence that participants in the included studies linked directly to their insecure status. The findings here suggest that gender is a significant marker of vulnerability to violence that is enhanced for women in insecure migration status specifically because of their insecure migration status, which is used by perpetrators to perpetuate power and fear. Nevertheless, we found a lack of research dealing specifically with migration-related violence experienced by a non-binary spectrum of gender identities. For this reason, the findings do not tell us whether or how violence might be enhanced for people who identify as trans and non-binary while in insecure migration status, nor does it suggest whether or how violence against male migrants might be specifically related to gender.

### Implications

Across the categories, violence against migrants in insecure status was not reported, or reporting was delayed, due to the risks associated with reporting or seeking protection in the form of immigration enforcement. Thus, to better protect migrants in insecure status from violence, victims of violence who report should be protected from immigration enforcement. This will remove an important form of insecurity that deters migrant victims from reporting violence. There should be a clear pathway to protection for people on spousal or employment visas who experience violence, that will not implicate their immigration status, and this should be available from the outset without the individual assuming any degree of risk that their immigration status will be compromised. The support services that are in place for citizens should be extended to people in insecure status who are victims of violence and abuse, regardless of their recourse to public funds. While this would not resolve all potential barriers to disclosure, it would address an important underlying insecurity connected to disclosing violence.

Violence during migration journeys, particularly violence perpetrated by the people migrants in insecure status rely on for border-crossing routes, is a key problem. While more research is needed in the context of successful initiatives to address this issue, it is the view of the authors that safe visa-free travel routes need to be made available to protect migrants without documents from harm.

### Strengths and limitations of review

This review was methodologically robust. We followed the PRISMA guidelines for the reporting of systematic reviews [33], and two reviewers were involved in screening, coding and extracting data from included studies, quality appraisal and interpretation. The qualitative thematic synthesis developed new themes that were not reported in the included primary studies. Nevertheless, this review is not exhaustive of all experiences of violence. We only included physical violence and sexual violence that was explicitly referenced as physical. Thus, it is likely we underreported violence that is inherent in threats and coercive action. Given that we note that migrants in insecure status often get trapped in violent situations that are prolonged or escalate, it would be revealing to include threats of violence and coercion (such as emotional, but also more practical via control of identity documents and finances) to better map how violence is associated with insecure migration status. We only included studies with direct quotes in the words of migrants in insecure status. Additional studies documented experiences in summary or stylised narrative form, or included insights from practitioners and support workers. We limited the review to peer reviewed academic literature. Indeed, qualitative evidence is often piecemeal, and a grey literature search may have yielded more results [31]. There could feasibly be forms of violence not documented by these studies, and relationships and patterns of violence that have not been identified in the limited sample. Nevertheless, while not describing a finite list of relationships and patterns of all types of violence associated with being in insecure migration status, this is the first qualitative systematic review of literature documenting experiences of violence where that violence is associated with insecure migration status. It offers important insight into the relationships and patterns of physical violence that are identified and observable here.

This systematic review raises the question of whether violence against women is overrepresented in the data because of the bias in included studies. More than half (54.5%) of included studies were of exclusively female participants. This could be for a number of reasons: for example, perhaps there is more violence against women, or maybe there is just more specified study of violence against women. It is also possible that the bias towards physical violence means women are overrepresented as victims and this obscures important forms of psychological or emotional violence linked with insecure migration status that is possibly more gender neutral or male biased.

### Diversity

The English language bias in the search and selection of studies means that violence in the US and UK is over-represented in the data. A more global geography of violence experienced by people in insecure status might be achieved by searching in multiple languages, or targeting specific languages linked to particular geographic areas determined by existing theoretical and empirical knowledge of migration routes and patterns. This review did not include the particular study of intersectional demographic characteristics and violence experienced by people in insecure status. Factors such as race, ethnicity, religion, cultural background, origin country, language spoken, place of residency, socioeconomic status, and age may intersect with experiences of violence.

### Recommendations for future research

Given the clear trend in sexual violence experienced by migrants in all types of insecure status, but especially along migration routes and journeys, there is a need for a targeted study of sexual violence along migration routes, sexual violence experienced by people in insecure status, and sexual violence experienced specifically by people with spousal and employment visas. There is an apparent relationship between the power imbalance introduced in dependent visa types such as employment and family visas, and more research should be carried out to understand the implications of this power imbalance with regard to all types of violence (physical, psychological, emotional, sexual). Preventing migrants from accessing public funds enhances this power disparity by increasing dependence on the relationship (whether with a partner or employer); this study suggests these policies need urgent revision.

While the studies included in this systematic review did not systematically report a range of demographic characteristics, to better understand patterns of violence linked to migration it would be necessary to review how these patterns are imposed on particular geographies or on preexisting inequalities. Therefore, we recommend further targeted research into intersectional experiences of insecure migration status and violence.

## Conclusion

This review found that people in insecure migration status experience physical and/or physically enforced sexual violence in ways directly linked with their insecure status; in ways linked to lacking access to support; and as a result of a fear of losing their status. These experiences were relevant globally, including data from seven regions and global migrants located in transit and destination countries. Experiences of violence were most frequently linked to being directly insecure, such as not having immigration status, being in undocumented status, or undertaking an irregular journey facilitated by smugglers or traffickers. Lacking recourse to support in order to leave violent circumstances was linked to prolonged experiences of violence or being trapped in violent situations. Fear of removal meant that people experiencing violence were reluctant to report that violence or to attempt to leave their relationship. This again meant that exposure to violence was prolonged over time. Sexual violence was reported most frequently, and other gendered dimensions of violence (such as violence escalating during pregnancy) were important in the experiences of people in insecure migration status. Migration journeys were often the sites of violence, and undertaking an undocumented journey involves a high risk of experiencing violence, especially sexual violence, and little recourse to protection or support in the event of experiencing violence.

## Electronic supplementary material

Below is the link to the electronic supplementary material.

Supplementary Material 1

Supplementary Material 2

Supplementary Material 3

Supplementary Material 4

Supplementary Material 5

Supplementary Material 6

## Data Availability

No datasets were generated or analysed during the current study.
