# Supplementary material for: Experiences of violence while in insecure migration status: a qualitative evidence synthesis
Source: Global Health. 2024 Nov 23;20:83. doi: 10.1186/s12992-024-01085-1 (PMC11585937; doi:10.1186/s12992-024-01085-1)
Supplement: Supplementary file 2 — Supplementary Material 2 [file 12992_2024_1085_MOESM2_ESM.pdf]

Appendix 2: Insecure Migration Status

**Immigration status**	**Examples of insecurity and related precarity (not finite)**
No status / undocumented	Subject to border enforcement (pushbacks, restraint, detention); subject to removal so tend to avoid contact with social services, police, healthcare where possible. Gives rise to vulnerability to exploitation in employment and living circumstances.
Asylum seeker	Subject to deterrence policies of the state. Subject to violence in detention facilities, restraint, removal. Subject to violent border policies, pushbacks.
Failed asylum seeker	As above, also subject to destitution and removal to unsafe location. Lack recourse to health, social and specialist services.
Overstayer / lapsed status	Subject to removal, destitution, vulnerable to exploitation, lack recourse to health, social and specialist services.
Not fulfilling conditions	Subject to removal, destitution, vulnerable to exploitation. Some scope to regain full visa by fulfilling conditions.
Change in circumstances	Subject to removal, destitution, vulnerable to exploitation. Some scope to regain full visa by filing change in circumstances.
No recourse to public funds	Enhances vulnerability to exploitation by the person to whom visa is tied (such as spousal). Leaving unsafe circumstances produces vulnerability to destitution.
Status tied to employer	Subject to enforced servitude, unsafe working conditions, unsafe living conditions with no recourse to report.
Status tied to family relationship	Vulnerable to domestic violence, enforced servitude, financial abuse, bureaucratic abuse.

Innes A. Migration, Vulnerability, and Experiences of Insecurity: Conceptualising Insecure Migration Status. Soc Sci. 2023 Oct;12(10):540.
