# Supplementary material for: Experiences of violence while in insecure migration status: a qualitative evidence synthesis
Source: Global Health. 2024 Nov 23;20:83. doi: 10.1186/s12992-024-01085-1 (PMC11585937; doi:10.1186/s12992-024-01085-1)
Supplement: Supplementary file 4 — Supplementary Material 4 [file 12992_2024_1085_MOESM4_ESM.pdf]

Paper Eligibility Criteria

Prevalence of violence experienced by people with insecure immigration status, and perceptions of association with immigration control.

In what ways do people in insecure migration status experience violence?

How is the violence experienced by people in insecure migration status related to their insecure status?

PEO: Participants = migrants / immigrants; Exposure = insecure immigration status; Outcome = violence. Phenomenon of interest = ways in which violence is linked to insecure migration status.

	**Yes – include**	**No – exclude**
**Study Type**<br>Is the study original empirical research published in a peer reviewed journal?	Peer-reviewed academic journal articles.	Book reviews<br>Reports<br>Conference papers<br>Systematic reviews (but use for additional source articles)<br>Conceptual or theoretical development with no empirical study<br>Magazine articles<br>Editorial<br><br>Reason: WRONG STUDY TYPE
**Source / Data Type**	Qualitative research documenting experiences during or after migration while a person is in insecure status.<br>First hand interview or ethnographic data from people who have experienced violence while in insecure status.<br>Second hand data gathered through interviews or ethnographic methods with professionals who	Theoretical or conceptual development with no data<br>Studies where data is not disaggregated by insecure migration status as defined above (eg 'foreign born' is not evidence of insecure status).<br>Studies where exploring experiences of violence while in insecure migration status was not a central focus of the study.

	Yes – include	No – exclude
	work with large populations of people in insecure status.<br>Administrative narrative data about people in insecure migration status (eg. court transcripts).	Reason: WRONG STUDY TYPE
**Participant Population**<br>People in insecure migration status at the time of experiencing violence<br>Practitioners who work with direct contact with people in insecure migration status.<br>?	People (of any age):<br>without any current immigration status (having left their country of citizenship or habitual residence);<br>or in the midst of an application for asylum or refugee status or another protected status;<br>or whose status has lapsed or who have overstayed a visa;<br>Or who have refugee status or protected status in a country where their permanent right to remain is not guaranteed in this status;<br>who have not continued to fulfil all of the conditions of their status;<br>or whose status has changed but they have not informed the immigration authorities or applied to change it;<br>or who are in a status that does not reflect their current circumstances;<br>or with No Recourse to Public Funds (UK) or the equivalent in other jurisdictions;<br>people who have a particular condition tied to their visa (such as being tied to a particular employer or family relationship such as spousal);<br>or who are otherwise under threat of removal.	Dual citizens who are residing in a country of their citizenship under conditions that do / did not threaten that citizenship at the time of experiencing violence;<br>lawful permanent residents who are living in conditions that do / did not threaten their status at the time of experiencing violence;<br>people who were not in insecure status when violence occurred (eg. Violence was experienced in home country as motivator of migration)<br>Mixed populations where it is impossible to extract data related to insecure migration.<br><br>Reason: WRONG POPULATION
**Phenomenon of interest**	There must be an observable relationship between the insecure status and violence.	Exclude studies where the relationship between insecure status and violence is not apparent.

	**Yes – include**	**No – exclude**
Perceived association/Relationship between insecure migration status and violence	For example, intimate partner violence where immigration documents are used as a source of threat.<br>Violence during transit where people in undocumented status have no recourse for protection.<br>Violence by an employer when a visa is tied directly to that employment.	For example, studies where violence is attributed to 'culture' and no association with status is made.<br><br>Reason: WRONG EXPOSURE
**Findings**<br>Interpersonal violence<br>**Individual's views, experiences.**	*Direct interpersonal violence* including things such as assault, rape, sexual violence, torture.<br>*Policies that utilize physical coercion*, such as forcible restraint, force feeding, forced use of tranquilizers.	Studies of structural violence such as poverty, food insecurity, homelessness, destitution that do not include specific examples or measurements of interpersonal / physical violence.<br>Historical violence that occurred pre 1990.<br><br>Reason: WRONG OUTCOME
**Notes:**<br>Eligibility criteria = hierarchy – begin with study type; work downwards until exclusion<br>1st exclusion criteria reached must be recorded as reason for exclusion (wrong study type, wrong population, wrong exposure, wrong outcome)		
