# Supplementary material for: Experiences of violence while in insecure migration status: a qualitative evidence synthesis
Source: Global Health. 2024 Nov 23;20:83. doi: 10.1186/s12992-024-01085-1 (PMC11585937; doi:10.1186/s12992-024-01085-1)
Supplement: Supplementary file 5 — Supplementary Material 5 [file 12992_2024_1085_MOESM5_ESM.pdf]

Risk of Bias in Included Studies

Adapted from the Critical Appraisal Skills Programme (CASP) Checklist for Qualitative Research

	Was there a clear statement of aims?	Is a qualitative method appropriate?	Was the research design appropriate to address aims?	Was recruitment strategy appropriate to address aims?	Was data collected in a way that addressed the research issue?	Has relationship between research and participant been considered?	Were ethical issues considered?	Was analysis sufficiently rigorous?	Is there a clear statement of findings?	Is the research valuable?
Adeyinka-2023	**	**	*	*	**	**	**	**	**	**
Anitha2008	**	**	**	**	**	*	-	*	**	**
Anitha2010	**	**	**	**	**	*	-	*	**	**
Baird2014	**	**	**	**	**	‡	**	*	**	**
Bhatia2019	**	**	*	-	‡	‡	-	‡	*	**
Boyd2019	**	-	‡	‡	‡	‡	-	‡	**	**
Critelli+Yalim2020	**	**	**	**	**	‡	-	**	**	**
Erez+Bach2003	**	**	**	**	**	‡	-	*	**	**
ErezEtAl2009	**	**	**	**	**	**	*	*	**	**
Fennig+Denov2022	**	**	*	**	**	*	**	**	**	**
GabreyesusEtAl2018	**	**	**	**	**	*	**	**	**	**
GabreyesusEtAl2019	**	**	**	**	**	‡	**	**	**	**
Grossman-Thompson2023	*	**	*	-	*	-	-	-	**	*
InfanteEtAl2020	**	**	**	**	*	‡	**	**	**	**

Jimenez-LasserrotteEtAl2020	**	**	**	-	**	‡	**	**	**	*
KeygnaertEtAl2014	**	**	**	**	**	**	**	**	**	**
KovnerEtAl2021	**	**	-	-	*	‡	-	*	**	**
LaughonEtAl2022	**	**	**	**	*	-	**	**	**	**
Leyva-Flores-2019	**	**	**	**	**	‡	**	*	**	**
Liang-2023	**	**	**	**	-	-	-	**	**	**
Liversage2021	**	**	**	**	**	‡	*	**	**	**
McMahon+Sigona2021	**	**	**	**	**	-	**	-	**	**
Minaye2012	-	**	**	-	**	*	-	**	**	-
Omar2022	**	**	**	-	**	**	-	‡	*	**
Pan+Yang2012	**	**	*	**	*	-	-	**	*	**
Parson-2016	**	**	**	-	**	‡	**	**	**	**
Radziwinowiczowna2020	**	**	*	-	-	‡	-	-	**	**
Reina+Lohman2015	**	**	**	**	**	‡	-	**	**	**
Reina-2014	**	**	**	**	**	**	-	**	**	**
Salcido+Adelman2004	**	**	**	**	**	-	**	-	**	**
Sharma 2017	*	-	-	-	-	-	-	-	*	*
ValdovinosEtAl2021	**	**	**	**	**	**	**	**	**	**
Vidales2010	**	**	**	**	**	*	-	**	**	**

**criterion completely satisfied; *criterion partially satisfied; - unclear or insufficient information; † criterion partially not satisfied; ‡criterion not satisfied; N/A Not applicable
