# Supplementary material for: Experiences of violence while in insecure migration status: a qualitative evidence synthesis
Source: Global Health. 2024 Nov 23;20:83. doi: 10.1186/s12992-024-01085-1 (PMC11585937; doi:10.1186/s12992-024-01085-1)
Supplement: Supplementary file 6 — Supplementary Material 6 [file 12992_2024_1085_MOESM6_ESM.pdf]

Appendix 6: Descriptive Codes

Deductive descriptive codes

1DIRECTINSECURE	**Exposure to violence (perceived) as a direct result of insecure status.** This code was applied to excerpts where the reason for the violence was directly related to insecure migration status with no other intervening factors. For example, violence that was experienced during a migration journey because of the state of being on the journey (e.g. violence from smugglers) was coded with this code. Violence that was experienced in the destination as a direct result of insecure migration status was coded with this code. Violence experienced by police and immigration enforcement during acts of immigration enforcement was coded with this code.
2FEAR-REMOVAL	**Prolonged exposure to violence because of fear of removal.** This code was applied to all excerpts where violence was happening because the victim was in insecure status and associated their experience of violence with a fear of immigration removal.
3LACK-RECOURSE	**Prolonged exposure to violence because of lack of recourse to state support (e.g. law, refuge, economic).** This code was applied to all excerpts where the person in insecure migration status cited that they did not qualify for state help and therefore could not leave their violent situation.
4GENDER	**Where experiences of violence associated with insecure migration status are linked specifically to gender (women).** This inductive code was applied to all excerpts where the violence was against women migrants in insecure status and the violence was specifically linked to their gender. This included cases of rape and sexual violence, but also other cases of gender-based violence.

Inductive Descriptive Codes

Code
1. CommunityViolence
2. employment-based-violence
3. family-violence
4. fear-of-child-protection
5. gender-sexuality-based-violence
6. in-destination-highly-vulnerable
7. in-transit-highly-vulnerable

8. IPV-unable-to-leave
9. NoAccessToSupport
10. Racist-violence
11. sexual-violence
12. Social-isolation
13. state-violence
14. stress-as-provocation
